# Nicotine pouches: a narrative review of the existing literature

**DOI:** 10.3389/fpubh.2025.1641308

**Published:** 2025-08-26

**Authors:** Hajed M. Al-Otaibi, Malik A. Althobiani

**Affiliations:** ^1^Department of Respiratory Therapy, Faculty of Medical Rehabilitation Sciences, King Abdulaziz University, Jeddah, Saudi Arabia; ^2^Respiratory Therapy Unit, King Abdulaziz University Hospital, King Abdulaziz University, Jeddah, Saudi Arabia

**Keywords:** non-combustible nicotine, smokeless, nicotine pouches, oral cavity, tobacco and tobacco product, smokeless carcinogens

## Abstract

**Introduction:**

Nicotine pouches are an emerging a non-combustible nicotine product. These tobacco-free oral products deliver nicotine through the gums. They are are viewed as potential alternatives to traditional tobacco and are marketed as safer alternatives.

**Aim:**

This review evaluates the health-related effects, explore consumer behavior, understand usage trends, and consider regulatory perspectives of nicotine pouches, focusing on their potential role in harm reduction.

**Results:**

Nicotine pouches contain variable levels of nicotine (1–47 mg/pouch), with high pH values (median 8.8) that may increase nicotine bioavailability through the oral mucosa. Although they contain fewer harmful substances than traditional tobacco products, some types do contain harmful chemicals including formaldehyde, chromium, and tobacco-specific nitrosamines, which raise concerns about potential long-term health risks. Limited preliminary studies suggest reduced toxicant exposure compared to combustible tobacco, but the long-term health effects are still unknown. Consumer awareness significantly varies (7–47%) across populations, with higher usage noted among males, younger adults, and former tobacco users. On a global level, regulatory approaches differ, from total bans in some countries to minimum age requirements in others.

**Conclusion:**

Nicotine pouches are quickly becoming a popular alternative to traditional tobacco, providing potential harm reduction by containing fewer harmful constituents. However, they still contain the addictive substance nicotine and, in some cases, trace amounts of substances such as formaldehyde, which have been detected at very low levels, often near or below detection limits, and may not be considered a significant health risk according to some studies. Although they may aid existing smokers in harm reduction, there are still concerns about youth initiation, dual-use, marketing practices, and long-term oral and cardiovascular health effects. Inconsistent regulations, variable product quality, and a lack of longitudinal studies indicates a need for comprehensive research to guide evidence-based policies. Policymakers should prioritize standardized testing and marketing controls to safeguard public health, adopting a precautionary approach until long-term safety is established.

## Introduction

Non-combustible nicotine-containing products have evolved in recent years, with nicotine pouches emerging as a new category within the smokeless, tobacco-free market (1, 2). These products first became available in Europe, the USA, and Japan around 2016 (3–5). Nicotine pouches, designed for oral use, differ from other oral products due to their unique composition and design. These pouches are typically made from a cellulose matrix infused with nicotine, which can be derived either from tobacco leaves or synthetically created (6). Flavorings, plant-based fibers, and other additives contribute to the functionality and appeal of these products (3). These features make nicotine pouches not only accessible but also competitive in the evolving market of nicotine delivery systems.

Nicotine pouches are usually placed between the gum and the lip, which allows for the gradual absorption of nicotine through the oral mucosal membrane (7). This method provides a discreet and convenient option for nicotine consumption without combustion, smoke, or residual odors generally associated with traditional tobacco products (3). Their lightweight design, coupled with a wide array of flavors and varying nicotine concentrations, makes them an accessible alternative for consumers seeking nicotine delivery methods other than smoking or other smokeless tobacco products like snus (8). However, despite the claimed benefits, concerns about potential health-related consequences and regulatory challenges have arisen. The currently available evidence regarding their safety and long-term health risks is insufficient, leaving a gap in the understanding of their overall impact on health.

Nicotine pouches are marketed as a tobacco-free nicotine product and are available in a white, nicotine-containing material, often in powder or moist cellulose based form (3). Manufactures often promote nicotine pouches as safer alternatives to traditional tobacco products, using terms like “Tobacco-free” or “Tobacco leaf-free” to imply they pose fewer health risks. This marketing may mislead adolescents and novice users about the safety of these products (9–11). The affordability of nicotine pouches contributes to their widespread use, making them an economical alternative to other tobacco and nicotine products (12–14). While the presence of harmful substances like carcinogenic nitrosamines complicates the risk assessment of these products, studies have found that the levels of these toxicants are often significantly lower than in combustible cigarettes and can be comparable to or lower than those found in nicotine replacement therapies (15, 16). The appeal to youth, enhanced by the availability of flavored options, increases the risk of addiction and that they could potentially serve as a gateway to conventional smoking (17, 18).

Current research, while contributing to our understanding may sometimes lack comprehensive long-term assessments (19–22). This scarcity of non-industry-funded studies creates an urgent need for independent research to assess their safety, usage patterns, and public health implications (15, 23, 24). This narrative review summarizes existing evidence on the use of nicotine pouches, focusing on health risks, consumer behavior, usage trends, and regulatory perspectives. By evaluating characteristics that distinguish nicotine pouches from traditional nicotine delivery systems, this review also explores their potential role in harm reduction efforts and highlights the critical gaps in knowledge requiring further investigation.

This narrative review was conducted through a comprehensive literature search using PubMed and Google Scholar. Key search terms included general phrases such as “nicotine pouches,” “oral nicotine products,” “tobacco-free nicotine pouches,” “smokeless nicotine,” “alternative nicotine delivery systems,” and “modern nicotine products,” as well as brand-specific terms like “DZRT,” “Velo,” “Zyn,” “On!,” “White Fox,” “Killa,” “Après,” “Pablo,” and “Klint.” We selected English-language articles that provided information about the safety, consumer behavior, regulatory framework, and market trends of nicotine pouches.

## Results and discussion

### Composition and potentially harmful substances in nicotine pouches

The source of nicotine in nicotine pouches, whether natural or synthesized is not always disclosed by manufacturers, though regulatory bodies like the FDA now require this information in premarket applications. Natural nicotine is extracted from the tobacco plant through a specific process. This method often introduces minor impurities and the presence of tobacco-specific nitrosamines (TSNAs). Conversely, synthesized nicotine is pure nicotine produced in a laboratory through chemical reactions, while typically free from TSNAs, its purity depends on manufacturing controls, and contamination is possible if production is not tightly controlled. The base material for nicotine pouches is called cellulose, which is made from plant fibbers to provide and maintain the pouch shape during use. Salt is commonly added to enhance nicotine absorption through the oral mucosal membrane and improve its taste. Also, xanthan gum might be used to maintain the texture of the pouch (25). Nicotine pouches are sold in a variety of fruits and flavors (3). Most nicotine pouch ingredients enhance the delivery of nicotine through the mucosal membrane and increase the product's appeal.

Several studies have analyzed the composition of nicotine pouches, including their weight and the pH of their extracts. It was found that the nicotine content varied extensively across different products. For instance, Stanfill et al. analyzed 37 samples from six distinct manufacturers and reported nicotine levels fluctuating between 0.89 and 6.73 mg per pouch (8). On the other hand, Malloch et al. examined 46 samples from 20 manufacturers and discovered that the nicotine levels per pouch ranged from 1.79 to 47.5 mg, with a median concentration of 9.48 mg/pouch (15).

The pH of nicotine products is a key determinant of the overall pharmacokinetic properties of nicotine. It has been demonstrated to augment its absorption and its physiological effect (26). Nicotine is an alkaline alkaloid with a pKa value of 8.01. At this value, half of the nicotine molecules are protonated and the other half remains unprotonated. As the pH of the nicotine product elevates, the percentage of unprotonated nicotine molecules also rises. This facilitates a greater amount of free nicotine to be available for absorption through the mucosal membrane (26–28). Therefore, the alkalinity of nicotine ensures it is easily absorbable through the oral mucosal membrane and becomes more readily available in the bloodstream at a faster rate (26, 29). Early studies revealed that the pH of nicotine pouches ranged from 6.94 to 10.4 (8), these values generate a nicotine proportion within the free nicotine range from 7.7% to 99.2%. Recent research reported a median pH (IQR) value of 8.8 (8.2–9.8), calculating the proportion of free nicotine to show a median of 86% (15). Manufacturers of nicotine pouches commonly aim to increase product alkalinity by adding alkaline agents such as carbonates and bicarbonates (28).

### Product manufacturing, marketing, design, and market growth

**Figure 1:**
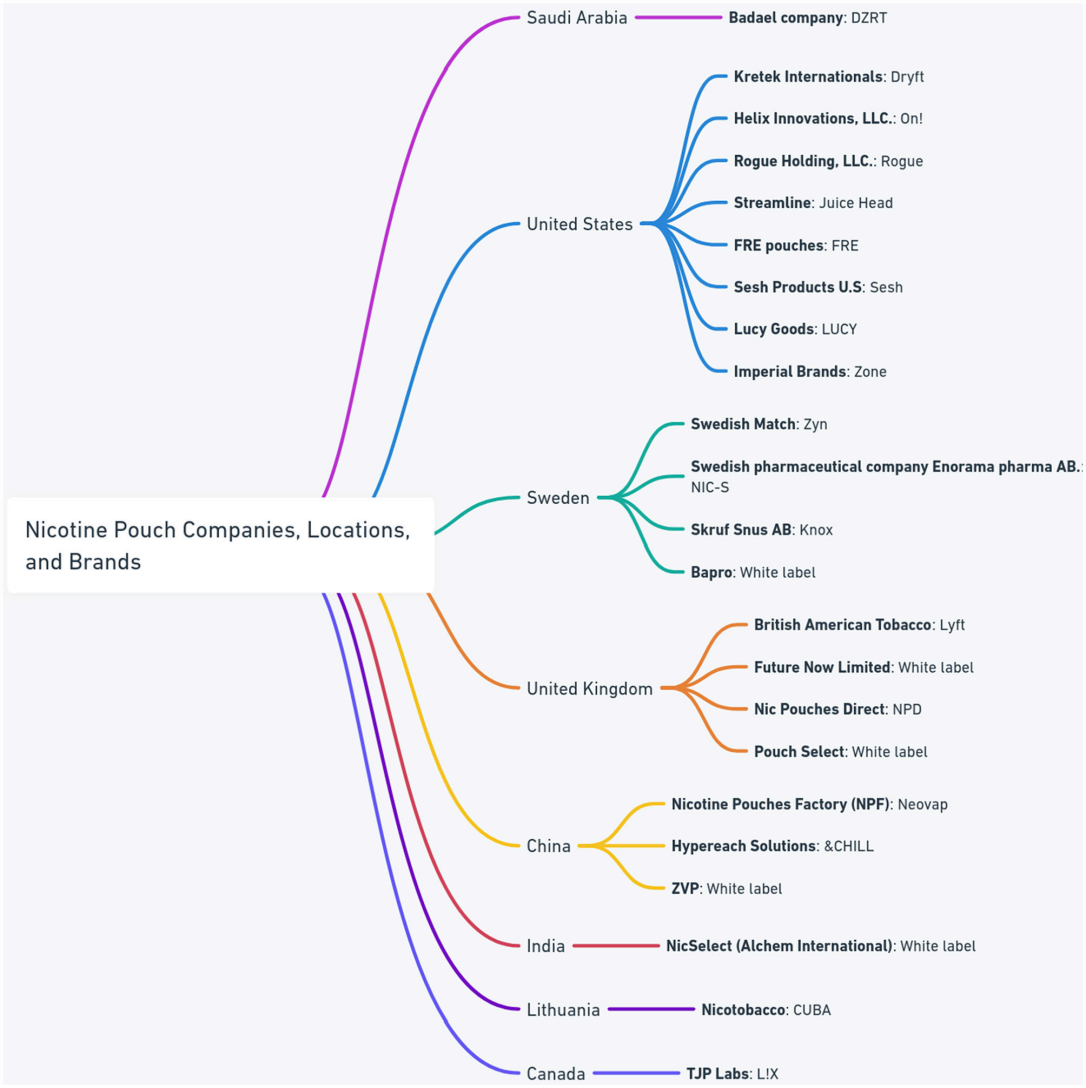
Major manufacturers of nicotine pouches and their global locations.

At present, an increasing number of manufacturers worldwide are introducing nicotine pouches to the market. Figure 1 presents a comprehensive overview of the global nicotine pouch market, showing the diversity of major manufacturers, their brands, and their geographic origins. The figure shows a widespread international presence, with companies operating from different regions including Saudi Arabia, the United States, Sweden, the United Kingdom, China, India, Lithuania, and Canada. Notably, the presence of “White label” products suggests a dynamic market where manufacturers may supply products for rebranding, indicating complex supply chains and market strategies within the industry.

**Figure 2:**
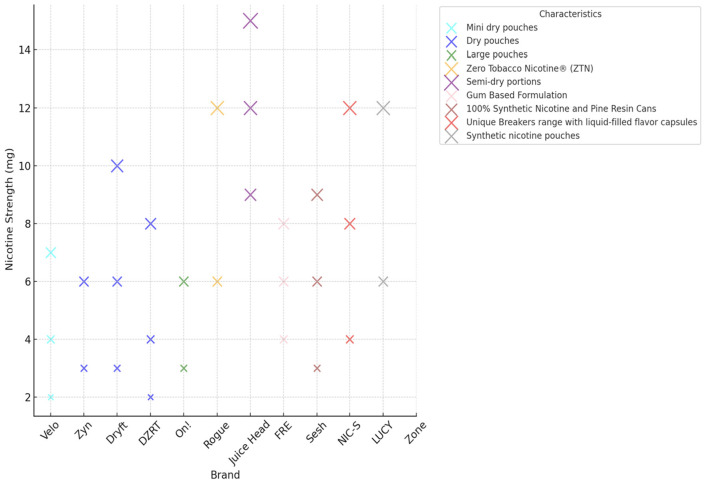
Nicotine characteristics and concentrations by brand.

These products are produced with varying nicotine concentrations and are available in a wide range of flavors such as mint, wintergreen, peppermint, citrus, fruit, coffee, cinnamon, and spearmint. These flavors enhance their appeal to diverse consumer groups. Figure 2 illustrates the wide range of nicotine strengths across different pouch brands, from as low as 2 mg to nearly 15 mg. For example, Juice Head products sit at the highest end of this scale, while brands like Velo and Zyn typically offer lower concentrations between 2 mg and 7 mg. Other brands, such as Dryft and FRE, also provide a diverse mix of strengths. This variety clearly shows how manufacturers are catering to different consumer preferences.

Between 2016 and mid-2020, the North American nicotine pouch market was led by five major manufacturers: Swedish Match, Altria, Kretek International, British American Tobacco, and Rogue. It expanded from 163,178 units, equivalent to US $709,635, in 2016, to 45,965,455 units, or US $216,886,819, by mid-2020. This growth signifies an average monthly percentage change (AMPC) of 10.9 (95% CI, 10.4–11.4) (30). The sector's rapid global growth has enabled new manufacturers. For example, Badael Company, which was established in Saudi Arabia in 2023, has introduced a new product named DZRT.

### Regulatory approaches and classification

Globally, nicotine pouch classification varies across countries, reflecting diverse regulatory frameworks and public health priorities. In the United States, for example, nicotine pouches are classified as tobacco products because they contain tobacco-derived nicotine (31). A recent international survey involving 67 countries revealed that nicotine pouches are available in nearly half of these nations (32), with 20 already implementing regulations. These regulations range from complete bans to requirements for minimal purchase age, warning labels, or advertising restrictions, indicating varying strategies to mitigate health risks (33).

On January 16, 2025, the US FDA authorized the marketing of 20 specific ZYN nicotine pouch products under the Premarket Tobacco Product Application (PMTA) process, marking the first such approval for nicotine pouches in the US (FDA Authorizes Marketing of 20 ZYN Nicotine Pouch Products). The FDA determined that these tobacco-free products pose a lower risk of cancer and other serious health conditions than combustible tobacco, benefitting adult smokers aged 21 and over as a harm reduction option. However, they remain addictive and not risk-free. The decision has faced criticism from the American Lung Association, warning that flavored variants like ZYN Citrus and Cool Mint could appeal to youth, potentially increasing youth tobacco use (American Lung Association: FDA's Authorization of Flavored Zyn is a Gift to Big Tobacco) (34). This indicates a need for ongoing research and strict youth access measures (35, 36).

**Figure 3:**
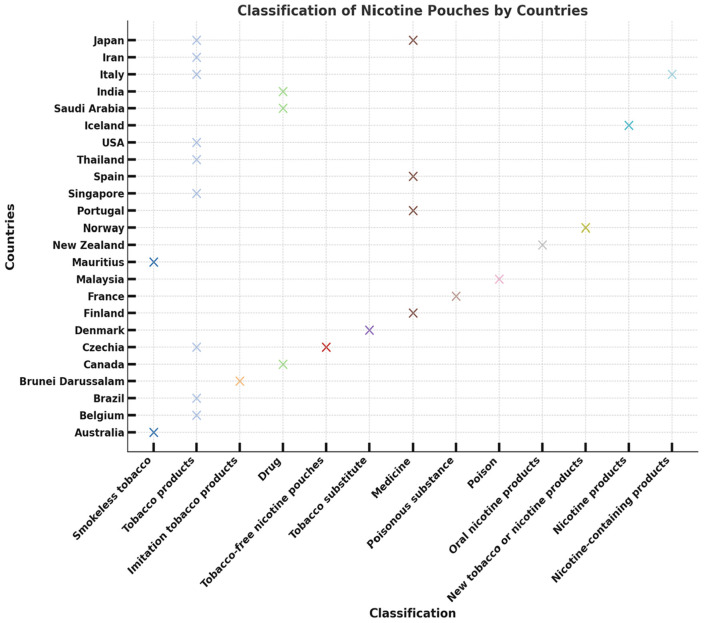
Classifications of nicotine pouches by countries.

Mitigation strategies might include prescription requirements for higher-dose products, outright prohibition (as in Singapore, Australia, and Brunei Darussalam), and the classification of synthetic nicotine pouches as pharmaceutical or drug products rather than traditional tobacco products. However, 13 of the surveyed countries where nicotine pouches are reportedly sold currently lack any regulatory framework (33). Figure 3 shows how the classification of nicotine pouches varies widely from country to country. For example, USA, Brazil, and Australia group them with “Tobacco products” or “Smokeless tobacco,” while other countries such as France, Japan, and Saudi Arabia define them more strictly as “Drug.” The classifications can be even more distinct, with Iran labeling the pouches as a poisonous substance and Italy designating them as nicotine products. This disparity clearly shows the lack of a unified global approach to regulating nicotine pouches.

### Health effects and safety considerations

The diverse ingredients in nicotine pouches, including nicotine, flavorings, sweeteners, pH adjusters, and plant-based fibers, introduce complexities regarding their short and long-term health risks. Nicotine is a highly addictive chemical, especially for adolescents whose brains are still developing and are known to be more susceptible to developing addiction (37). Studies have shown that nicotine has detrimental effects on brain development, a process that continues until approximately age 25 (32). Using nicotine during this critical period can impair the development of brain regions responsible for learning, mood regulation, memory, attention span, and impulse control (38). In addition, early exposure to nicotine can increase an adolescent's risk of developing future addictions to other drugs (39). Potential immediate physiological responses to nicotine pouches include an increase in heart rate, blood pressure, feelings of dizziness, nausea, irritation, headaches, difficulty sleeping, dry mouth, soreness, and a burning sensation in the mouth (40). Prolonged use of nicotine carries potential long-term risks, especially concerning the cardiovascular system (41). Sustained exposure to nicotine can strain the heart and blood vessels, potentially increasing the likelihood of developing hypertension, heart disease, heart attack, and stroke. Nicotine's vasoconstrictive properties can narrow arteries and may contribute to the process of atherosclerosis (42). Also, the direct and prolonged contact of nicotine and other chemicals within the pouches with the oral mucosa presents a persistent risk for various long-term oral health issues (4, 43).

Nicotine pouches generally seem to contain fewer harmful constituents than cigarettes and certain smokeless tobacco products (12, 29, 39–41). However, formaldehyde, chromium, ammonia, nickel, and tobacco-specific nitrosamines (TSNAs) have been detected in some formulations at levels comparable to or exceeding those in snus, nicotine replacement therapies (NRTs), in contrast, are pharmaceutical grade and subject to stricter purity standards (12, 26, 29, 39, 40). *In vitro* analyses have shown various degrees of cytotoxic and inflammatory responses across different nicotine pouches brands, possibly influenced by flavorings (12, 26, 29, 39, 40, 42). Also, 2021 data reported that nicotine pouches contain chromium and formaldehyde compounds at a quantifiable level (6). Both chromium and formaldehyde are classified as harmful and potentially harmful products (HPHCs). According to the authors, the level of formaldehyde is similar to that in smokeless tobacco (snus) but higher than in nicotine replacement therapy (NRT). Nevertheless, the levels of cadmium, arsenic, acetaldehyde, lead, and nitrosamines NDMA, NNK, and NNN are lower than in snus (6). Similarly, Mallock et al. were able to detect nitrosamines in 26 out of 47 nicotine pouches tested (15). An independent analysis of 48 nicotine pouches from 22 manufacturers identified 186 distinct chemicals, including eight classifieds as hazardous by the European Classification, Labeling, and Packaging Regulation. We also found that, three substances (methyl eugenol, benzophenone, β-myrcene) have been identified as potentially carcinogenic by the International Agency for Research on Cancer (18).

### Consumer perceptions and knowledge gaps

Awareness of nicotine pouches varies greatly among different populations, ranging from 7% among Dutch adolescents and adults to 47% among current or former adult tobacco users in the United States (44–48). Across numerous studies, it has been observed that males, younger adults, and those with a history of smoking or vaping are more likely to use nicotine pouches. Awareness among young adults exceeds 40%, particularly among individuals who have previously or currently use cigarettes, cigars, e-cigarettes, or smokeless tobacco (44–48). National surveys conducted in Poland, the United Kingdom, and the United States also suggest that younger adults, men, and individuals with a history of smoking or vaping are more likely to be aware of nicotine pouches (44–49). Despite the increasing awareness, the overall prevalence of nicotine pouches use remains relatively low in most surveyed populations, with rates of ever-use ranging from 9% to 13% (Poland, the UK, and the US), and current use around 2% to 4% (44–46, 48). In a selected sample of young Australians, 26% reported having ever used nicotine pouches and 19% had used them in the past 30 days, indicating higher rates in certain demographics and groups (47). However, prevalence should be linked to product availability in specific markets, particularly given that Australia introduced strict tobacco control laws in 2025 aimed at reducing youth smoking initiation (50).

Consumer understanding of nicotine pouches varies significantly, as many people are uncertain about their potential risks and benefits. Some individuals perceive nicotine pouches as less hazardous than cigarettes but remain unclear on how they compare to other smokeless tobacco products. In contrast, others believe that nicotine pouches could potentially be equally or even more harmful than combustible cigarettes (44–48). Marketing labels, such as “tobacco-free,” can diminish perceived harm and increase interest, especially among the youth and non-tobacco users (44, 48). Common reasons for experimenting with nicotine pouches include an attempt to quit or reduce smoking or vaping, the avoidance of odor, and a curiosity about the flavors or the “buzz” (51–53). Nonetheless, misconceptions about the role of nicotine in causing smoking-related harms continue to persist (44–48).

**Figure 4:**
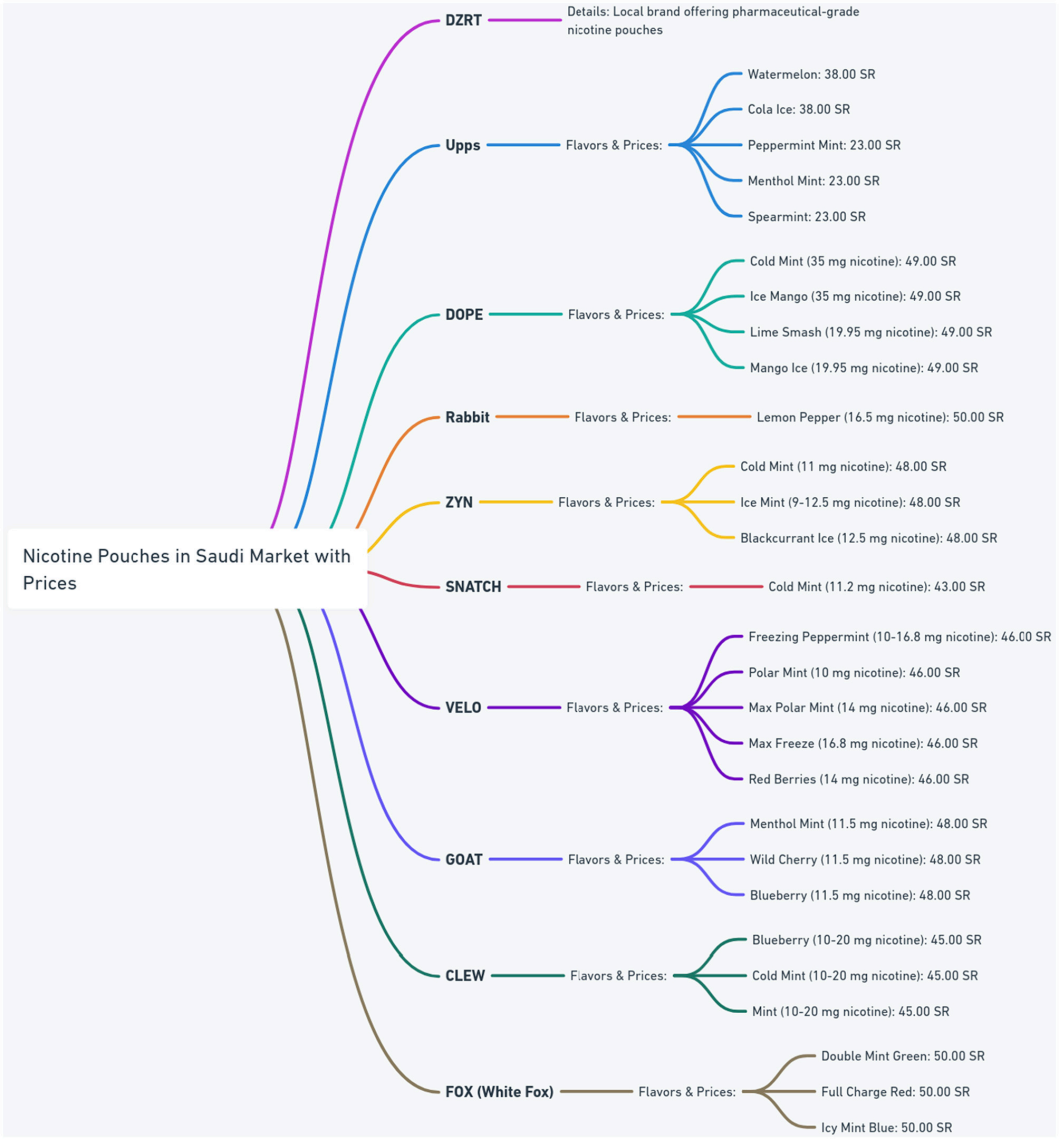
Nicotine pouches in the Saudi market.

The nicotine pouch market in Saudi Arabia is experiencing rapid growth, as shown by a diverse range of brands in Figure 4. The figure provides a detailed overview of the nicotine pouch market in Saudi Arabia, showing different brands, their available flavors, nicotine strengths, and corresponding prices in Saudi Riyals (SR). The figure highlights a diverse range of products, with brands such as Upps offering multiple mint and fruit flavors at prices around 23.00–38.00 SR. Other brands like DOPE and Rabbit feature higher nicotine concentrations, with DOPE offering 35 mg options for 49.00 SR and Rabbit providing 16.5 mg for 50.00 SR. ZYN and SNATCH present options with varying nicotine levels and prices, while VELO offers a broad spectrum of nicotine strengths (10–16.8 mg) at a consistent price of 46.00 SR. GOAT and CLEW also contribute to the market diversity with their own flavors and strength profiles. DZRT is identified as a local brand offering pharmaceutical-grade nicotine pouches, suggesting a unique positioning in the market. This figure shows the competitive market and the wide array of choices available to consumers in Saudi Arabia.

Concerns have arisen regarding the quality and distribution practices of international brands such as Upps, DOPE, ZYN, VELO, GOAT, CLEW, and FOX (White Fox). Many of these are sold through third-party and online retailers. The absence of direct oversight from the manufacturers of these brands raises several issues. Third-party online sales might lack rigorous regulation, leading to inconsistent nicotine concentrations, misleading labeling, and easier access for minors who can bypass age verification. The lack of regulation and manufacturer oversight also increases the threat of counterfeit products which compromise quality and pose health risks (54–56).

To promote a responsible nicotine pouch market and minimize public health risks, manufacturers should adhere to pharmaceutical-grade production standards. These measures guarantee quality and safety, thus reducing risks associated with counterfeit or substandard products. Distribution should be confined to regulated channels with stringent age verification mechanisms to prevent underage access. Also, transparent practices such as accurate labeling of nicotine strength and ingredient information are essential for informed consumer choice. While safety measures are essential, they do not eliminate the inherent health risks associated with nicotine pouches themselves.

Over a decade ago, the harm reduction continuum was proposed (32, 57). It systematically classifies nicotine-containing products based on their potential harmful effects. It categorizes all combustible tobacco products as the most harmful while presenting non-combustible nicotine products as the least harmful. As such, the continuum encourages transitioning from more harmful products to less harmful ones (32). Preliminary clinical studies suggest potential oral health benefits and reduced biomarkers of exposure when switching from combustibles or SLT to nicotine pouches, though the long-term health impacts remain unclear (58–61). Also, concerns persist regarding dual-use and uptake by youths and nicotine-naïve individuals, particularly as nicotine pouches are marketed with “tobacco-free” messages and attractive flavors (5, 11, 30, 62–64). Considering the known risks associated with various tobacco products (65–67) and the potential for these pouches to either reduce harm or perpetuate nicotine dependence (68), further research, including longitudinal studies, will be essential to clarify the overall impact of nicotine pouches on public health, their toxicity profiles, cessation efficacy, and usage patterns (69–72). This review emphasizes the importance of clear legislation that addresses both tobacco-derived and synthetic nicotine, particularly for countries still ill-equipped to regulate emerging nicotine products. Policymakers in these countries can refer to examples from jurisdictions that have adapted their definitions of tobacco control legislation and incorporated all nicotine sources under drug and medicine regulations. This shift from a tobacco-centric model to one focusing on nicotine can ensure strong consumer protection, especially for vulnerable populations like youths. Usage patterns highlight the importance of targeted public health interventions to ensure that accurate information reaches these at-risk groups.

## Conclusion

This review reveals inconsistencies in nicotine levels, pH, and chemical profiles that differ widely among various nicotine pouch products. Some products contain hazardous substances such as formaldehyde, chromium, and nitrosamines, which may increase the risk of adverse health outcomes. Although these products might offer a less harmful alternative to combustible tobacco, their long-term safety remains uncertain. There are ongoing concerns about youth initiation, dual-use, misleading marketing practices, and potential long-term oral and cardiovascular health effects. Given the variable product quality and inconsistent regulatory frameworks, it is essential to conduct more rigorous and longitudinal research. This research should compare these products with other nicotine delivery systems, clarify their roles in cessation, and assess their broader public health impacts. Policymakers should prioritize standardized testing, transparent labeling, and strict marketing controls, adopting a precautionary approach until the long-term safety of nicotine pouches is firmly established.
